# HBV Infection in Relation to Consistent Condom Use: A Population-Based Study in Peru

**DOI:** 10.1371/journal.pone.0024721

**Published:** 2011-09-13

**Authors:** Antonio Bernabe-Ortiz, Cesar P. Carcamo, John D. Scott, James P. Hughes, Patricia J. Garcia, King K. Holmes

**Affiliations:** 1 School of Public Health and Administration, Universidad Peruana Cayetano Heredia, Lima, Peru; 2 Department of Medicine, University of Washington, Seattle, Washington, United States of America; 3 Center for AIDS and STD, University of Washington, Seattle, Washington, United States of America; 4 Departments of Global Health and Medicine, University of Washington, Seattle, Washington, United States of America; U.S. Naval Medical Research Unit Six (NAMRU-6), Peru

## Abstract

**Background:**

Data on hepatitis B virus (HBV) prevalence are limited in developing countries. There is also limited information of consistent condom use efficacy for reducing HBV transmission at the population level. The study goal was to evaluate the prevalence and factors associated with HBV infection in Peru, and the relationship between anti-HBc positivity and consistent condom use.

**Methods and Findings:**

Data from two different surveys performed in 28 mid-sized Peruvian cities were analyzed. Participants aged 18–29 years were selected using a multistage cluster sampling. Information was collected through a validated two-part questionnaire. The first part (face-to-face) concerned demographic data, while the second part (self-administered using handheld computers) concerned sexual behavior. Hepatitis B core antibody (anti-HBc) was tested in 7,000 blood samples. Prevalences and associations were adjusted for sample strata, primary sampling units and population weights. Anti-HBc prevalence was 5.0% (95%CI 4.1%–5.9%), with the highest prevalence among jungle cities: 16.3% (95%CI 13.8%–19.1%). In the multivariable analysis, Anti-HBc positivity was directly associated with geographic region (highlands OR = 2.05; 95%CI 1.28–3.27, and jungle OR = 4.86; 95%CI 3.05–7.74; compared to coastal region); and inversely associated with age at sexual debut (OR = 0.90; 95%CI 0.85–0.97). Consistent condom use, evaluated in about 40% of participants, was associated with reduced prevalence (OR = 0.34; 95%CI 0.15–0.79) after adjusting for gender, geographic region, education level, lifetime number of sex partners, age at sexual debut and year of survey.

**Conclusion:**

Residence in highlands or jungle cities is associated with higher anti-HBc prevalences, whereas increasing age at sexual debut were associated with lower prevalences. Consistent condom use was associated with decreased risk of anti-HBc. Findings from this study emphasize the need of primary prevention programs (vaccination) especially in the jungle population, and imply that condom use promotion might be a potential strategy to prevent HBV infection.

## Introduction

Globally, approximately 400 million people have chronic Hepatitis B virus (HBV) infection, the cause of more than 500,000 deaths worldwide annually [1]. The clinical spectrum of chronic HBV infection ranges from the asymptomatic carrier state to liver failure, chronic hepatitis, liver cirrhosis, and hepatocellular carcinoma [2].

HBV is transmitted through percutaneous or mucosal exposure to infectious blood or other body fluids [3]. Blood, semen and saliva have been found to be infectious [4]. Among adults, HBV transmission is associated primarily with sexual experience and injection drug use (IDU) [5].

Highest HBV prevalences occur in people with multiple sex partners, female and male sex workers, and sexual contacts of persons with HBV infection [6], [7], [8], [9]. Cross-study comparisons of HBV prevalences are complicated by the use of different serological markers. For example, many general population surveys are based on the prevalence of surface antigen (HBsAg) [10], whereas studies of high-risk populations including sexually transmitted diseases (STD) clinic patients, MSM and IDUs have more often assessed antibody against hepatitis B core (anti-HBc) and antibody against surface antigen (anti-HBsAg) [7], [8], [9]. Overall, the presence of HBsAg indicates current acute or chronic HBV infection, while anti-HBc is a marker of exposure to HBV, and anti-HBsAg represents antibodies acquired either from vaccine or natural infection [11].

In developing countries, data on prevalence of HBV infection in the general population are limited. Some studies have found HBsAg carriage more prevalent among men than among women [10]. In Peru, HBsAg seroprevalence has ranged between 0.82% and 5.2%, depending on the regions and populations evaluated [12], [13]. Recently, as a part of a large community randomized trial of STD prevention, the Peru PREVEN project, we analyzed sera from men and women aged 18 to 29 years for HBsAg. The overall prevalence in this study was 0.28% [14]. This lower prevalence may be explained by the involvement of only urban participants aged from 18 to 29 years, as well as the past implementation of HBV vaccination campaigns, especially in high-risk populations, which might have had an impact on the population-based prevalence.

Two previous studies of FSW have found that consistent condom use might reduce HBV transmission [15], [16]. There are limited data evaluating anti-HBc prevalence and its association with consistent condom use at the population level. The purpose of this study was to determine the prevalence of anti-HBc in relation to demography, risk behaviors and condom use using blood samples and behavioral data previously collected from random samples of young adults in 28 Peruvian cities.

## Methods

### Study setting and design

Data and sera from two different cross-sectional surveys carried out in mid-sized cities of Peru have been used for this study. The first survey was performed between September and December 2005, and the second between August and October 2007. In both surveys, data concerning sexual behavior as well as biological samples were collected using handheld computers and identical methodology.

The PREVEN study, a community randomized trial (CRT) of STD prevention, began in the year 2000, with development and testing of several strategies for STD prevention in Peruvian cities with more than 50,000 inhabitants. In the 2005 survey, 20 cities participated; while in 2007, the survey included 8 additional cities, including Lima, the capital of Peru. Originally, 600 participants (300 of each sex) were recruited per city. For this particular analysis, a random sample of 250 participants per city was analyzed.

### Study population and sampling methodology

The methodology of PREVEN population-based surveys has been previously described [17], [18]. Briefly, a random sample of men and women between 18 and 29 years was selected using a multistage cluster sampling. The first step involved a random sample of 108 clusters of contiguous residential blocks for each participating city (each block contained 40 households on average). Second, a census of households in the selected clusters was conducted by nurses or midwives trained in the sample frame methodology, in performing the interviews, and in obtaining biological specimens. They first collected general information about location and type of housing, number of people living in the household, including number of people between 18 and 29 years of age, and length of time residence in the city. Then, a random sample of 10 households with eligible members was chosen. Potential participants were male or female, aged 18-29 years, living in the city for at least 6 months. Finally, in selected households with one or more eligible person, a participant was selected based on most recent birthday.

Selected participants were then interviewed by the nurses and midwives to confirm eligibility, obtain informed consent and administer the questionnaire. The informed consent was oral to keep each participant's information anonymous, and was approved by the Institutional Review Boards of the University of Washington and Universidad Peruana Cayetano Heredia. The interviewer signed the form to document participant's approval.

This procedure was performed until a target sample size of 300 men and 300 women per city had been recruited.

### Demographic and sexual behavior data collection

Data were collected through a previously validated two-part questionnaire. The first part was administered face-to-face, eliciting data concerning demographic characteristics, employment, marital status, knowledge about STDs, and reproductive health. The second part was self-administered using handheld computers, and concerned sensitive characteristics of the participant's sexual behavior. Completing both parts took approximately 30 minutes. The face-to-face interview was conducted in the absence of third parties and the self-applied questionnaire was completed out of sight of the interviewer to ensure confidentiality.

### Collection and testing of biological samples

After the questionnaire was completed, the interviewer explained procedures for sample collection. Men were asked to provide urine while females were asked to provide self-obtained vaginal swabs. Females unwilling to provide vaginal swabs were asked to provide urine. Interviewers then collected venous blood samples from men and women using a vacuum extraction system (8–10 ml). Samples were transported to the local laboratory within 4 hours of collection. Serum was separated from the blood sample and then separated again into three 1-ml vials and frozen at −20°C. Cryovials were sent frozen to the Universidad Peruana Cayetano Heredia laboratory for storage and analysis.

Syphilis diagnosis was based upon positive RPR Nosticon II (Organon Teknika), with confirmation with Serodia-TPPA (Fujirebio, Inc). HIV diagnosis was based upon Uni-Form II Ag/Ab (Biomerieux-Vironostika) ELISA NEW LAV BLOTI (BIO-RAD) as a confirmatory test. All diagnostic tests were performed following manufacturers' procedures.

Antibody against HBc (anti-HBc) was tested by electro-chemo-luminescence immunoassay to determine in vitro the qualitative presence of immunoglobulin M and G against HBc in sera using a ROCHE Elecsys automatic analyzer. All of the process was performed in accordance with manufacturer's instructions. The lower limit of detection for this test is ≤0.8 Paul Ehrlich Institute units per ml (U-IPE/ml). Without confirmatory testing, the reported sensitivity is 100% and the specificity 99.6% [19].

### Sample size

During the 2005 survey, information was collected from 14,280 participants, of whom 11,825 (82.8%) provided blood samples and gave approval for sample storage and use for future studies. In the 2007 survey, data were collected from 5,921 participants, of whom 4,781 (80.7%) provided blood samples and approved sample storage and use for future studies. Due to budget limitations, 250 samples and their respective questionnaires for each city were randomly selected from all the blood samples collected in the 2005 and 2007 surveys, yielding a total of 7,000 serum specimens.

### Data analysis

Statistical analyses were performed using STATA 11.2 for Windows (STATA Corporation, College Station, Texas, US). Prevalences and associations were assessed taking into account sample strata, primary sampling units and population weights using SVY methods. Crude results of associations are shown in Supporting Information S1.

Initially, a brief description of participants and anti-HBc prevalence was calculated, as well as prevalences according to geographic regions and risk populations. Potential risk factors associated with anti-HBc prevalence were calculated using Pearson's Chi squared (χ^2^) test, Fisher's exact test and t-test for independent samples according to the variable analyzed. Bivariate models were obtained using logistic regression analysis. Variables found to be significant (p<0.10) in bivariate analyses were entered into a multivariable logistic regression model using a forward stepwise method to determine odd ratios (OR) and their 95% confidence intervals (95%CI).

Adjusted odd ratios (aOR) were then calculated using logistic regression estimates to measure associations between anti-HBc prevalence and self-reported condom use. The condom use variable was evaluated using detailed questions concerning the number of sexual encounters and condom use over the previous three months with each of the last three sex partners. For each of the last three partners, the respondent was asked “during the last three months with that partner, how many times did you have sexual intercourse?”, and was then asked “how many times did you have sexual encounters without using a condom?” In the analysis, consistent condom use was defined as reported condom use “always” during the last three months with each of the last three sex partners. In the same way, never use of condom was defined as “never” using condoms with any of the last three sex partners, and all other responses were classified as occasional.

All variables evaluated as potential risk factors were assessed as potential effect modifiers and/or confounders. Potential effect modifiers were assessed using logistic regression and likelihood ratio tests (comparing the model with and without the interaction term). Potential confounders were considered when the adjusted risk estimate differed from the crude estimate by 10% or more. Attributable risk percent (AR%), defined as the likelihood that the disease in a exposed person was due to exposure, was calculated as performed in case control studies assuming that RR is very similar to OR (i.e. rare disease) [20]. Thus, the AR% was calculated based on the OR of comparing odds of consistent condom users to odds of those who reported never use condom, according to the following formula:
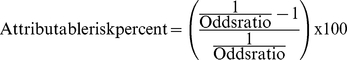



## Results

Seven thousand participants were included in this analysis. Of these, 3298 (47.6%) were men. The mean age was 22.9 (SD: 3.5) years, 57.3% of participants had less than 12 years of education and 64.2% were never married.

### Prevalence of HBV infection

Overall, the prevalence of anti-HBc taking into account samples strata, primary sampling units and population weights was 5.0% (95%CI: 4.1% –5.9%). Anti-HBc prevalence was 3.3% (95%CI: 2.2% –4.6%) in coastal cities, 5.9% (95%CI: 4.6%–7.4%) in the highlands cities, and 16.3% (95%CI: 13.8%–19.1%, p<0.001) in the jungle cities.

Using information from another assessment of HBsAg prevalence in a random sample of the same group of participants [14], a total of 4,356 participants had both anti-HBc and HBsAg results. Overall, of 324 anti-HBc positive participants, 15 (4.6%) were also HBsAg positive.

When prevalence was stratified by risk populations, anti-HBc prevalence was 10.1% (95%CI 4.4%–21.3%) among 341 men who reported ever having sex with men compared to 5.7% (95%CI 4.4%–7.3%) among 2,528 exclusively heterosexual men. Similarly, adjusted anti-HBc prevalence was 3.3% (95%CI 1.3%–7.9%) among 112 women reported ever having received money or other goods for sex compared to 4.4% (95%CI 3.5%–5.7%) among 2914 women who did not.

### Risk factors for HBV infection

Demographic variables associated with anti-HBc positivity (Table 1) included participant sex (p  =  0.009) and geographic region (p<0.001). Among sexual behaviors, younger age at sexual debut (p<0.001) was the only variable associated with anti-HBc positivity after taking into account sample design. The prevalence of anti-HBc among MSM was highest for those reporting both insertive and receptive anal sex; however, this trend was not significant (Table 2).

**Table 1:** Demographic variables associated with HBV positivity: percentages and p-values account for sample strata, primary sampling units and population weights.

Variables	Anti-HBc positive/Total	p-value
***Gende*** *r ^+^*		
Male	242/3298 (6.16%)	0.009
Female	266/3635 (4.13%)	
***Geographic region***		
Coastal	118/3250 (3.32%)	<0.001
Highlands	136/2000 (5.87%)	
Jungle	258/1750 (16.25%)	
***Educational level*** *^+^*		
≤11 years	329/3885 (5.25%)	0.32
>11 years	161/2894 (4.37%)	
***Age at interview*** *^+^*		
18–21 years	186/2805 (4.90%)	0.59
22–25 years	164/2226 (5.55%)	
26–29 years	158/1897 (4.55%)	
***Employment*** *^+^*		
Employed	187/2402 (5.24%)	0.66
Unemployed	318/4515 (4.88%)	
***Income*** **, +		
No incomes	251/3479 (5.20%)	0.69
1–500 NS	185/2538 (4.59%)	
>500 NS	62/800 (5.49%)	
***Marital status*** *^+^*		
Never married	285/4446 (4.78%)	0.46
Ever married	222/2479 (5.39%)	

+ Numbers may not add to the total because of missing values.

*Unless stated otherwise: percentages are calculated from row totals.

**NS = Nuevos Soles.

**Table 2:** Sexual behavior and STI variables associated with HBV positivity: percentages and p-values account for sample strata, primary sampling units and population weights^ +, ++^.

Variables	Anti-HBc positive/Total	p-value
***Age at sexual debut***		
<16 years	171/1589 (8.77%)	<0.001
16–24 years	256/4052 (4.02%)	
25–29 years	5/83 (3.57%)	
***Lifetime number of sex partner***		
0	60/980 (3.95%)	0.25
1–2	205/3140 (4.57%)	
3–4	104/1334 (5.55%)	
5 or more	116/1227 (6.57%)	
***Last year, new sex partners***		
0	227/3345 (4.79%)	0.17
1 or more	85/1066 (6.60%)	
***Ever sex with FSW*** **		
No	134/1834 (6.30%)	0.79
Yes	78/1035 (5.81%)	
***Ever sex with MSM*** **		
No	176/2513 (5.67%)	0.73
Yes, only insertive sex	8/73 (8.34%)	
Yes, only receptive sex	3/21 (3.99%)	
Yes, both types of sex	7/35 (7.97%)	
***Received money or other goods for sex (only females*** *)*		
No	228/2973 (4.43%)	0.53
Yes	4/53 (3.33%)	
***Syphilis***		
No	504/6953 (4.98%)	0.22
Yes	8/37 (10.71%)	
***HIV infection***		
No	509/6959 (5.02%)	0.57
Yes	3/14 (3.25%)	

+ Numbers may not add to the total because of missing values.

++ Analyses included to those with sexual activity (N = 5936) except for lifetime number of sex partners.

*Unless stated otherwise: percentages are calculated from row totals.

**Only male respondents.

In the multivariable analysis, anti-HBc positivity was independently associated with geographic region (greater risk in highlands and jungle compared to coastal cities) and younger age at sexual debut (Table 3).

**Table 3:** Risk factors for HBV infection taking into account sample strata, primary sampling units and population weights: bivariate and multivariate model.

Variables	Bivariate model	p-value	Multivariable model*	p-value
	OR (95%CI)		OR (95%CI)	
***Gende*** *r*				
Male	1 (Reference)	0.01		
Female	0.66 (0.48–0.90)			
***Geographic region***				
Coastal	1 (Reference)		1 (Reference)	
Highlands	1.82 (1.18–2.79)	0.006	2.05 (1.28–3.27)	0.003
Jungle	5.66 (3.79–8.44)	<0.001	4.86 (3.05–7.74)	<0.001
***Age at sexual debut §***				
Years (Mean)	0.88 (0.84–0.94)	<0.001	0.90 (0.85–0.97)	0.002

*Stepwise logistic regression analysis, adjusted for all demographic and sexual behavioral variables significantly associated with anti-HBc positivity in bivariate analyses (p<0.10).

^§^Per year of delay of first sexual intercourse.

### Self-reported condom use and HBV infection

Among the 2,874 (41.1% of the total) participants who responded to the questions about condom use, 13.9% reported consistent use, 42.5% occasional use, and 43.6% never used with any of the last three partners. Overall, 46.5%, 31.3% and 45.9% of participants reported never used condom in coastal, highlands and jungle, respectively. Anti-HBc prevalence was 2.6% among consistent condom users compared to 4.9% among occasional users, and 5.8% among never users.

After assessing variables analyzed in the first part of this study, no effect modifiers were found, whereas gender, geographic region and age at sexual debut were potential confounders. After adjusting for aforementioned variables, year of study, as well as education level and lifetime number of sex partners (confounders previously described in the literature), condom use was associated with anti-HBc positivity (adjusted Wald test's p-value = 0.03). Consequently, risk estimates were: aOR = 0.72 (95%CI 0.43–1.21) for occasional condom users and aOR = 0.34 (95%CI 0.15–0.79) for consistent condom users compared to never users (Table 4). Crude results and strength of association by geographic region are shown in Supporting Information S2. When estimates were calculated by geographic region, association was not significant. Although direction of the association was the same in the three sites, significance was not reached due to lack of power (See Supporting information S3).

**Table 4:** Association between self-reported condom use and HBV infection taking into account sample strata, primary sampling units and population weights.

Variables	HBV (−)	HBV (+)	Adjusted Model*
	n (%)	n (%)	OR (95%IC)
***Self-reported condom use***			
Never user	1059 (94.2)	105 (5.8)	1 (Reference)
Occasional user	1248 (95.1)	95 (4.9)	0.72 (0.43–1.21)
Consistent user §	343 (97.4)	19 (2.6)	0.34 (0.15–0.79)

§ Consistent condom users were those who reported condom use “always” during last three months with each of their sexual partners.

*Adjusted for gender, geographic region, education level, lifetime number of sex partners, age at sexual debut, and year of survey.

Based on these results, it was possible to calculate the attributable risk percent of consistent condom use: about 66% of the HBV infected among non-condom users was due to never using condoms. Similarly, we calculated the attributable risk percent for occasional condom users: 28%, although adjusted model shown a non-significant estimate compared to non-users.

## Discussion

In this report of a large and representative sample, we report anti-HBc prevalences and sexual factors associated with anti-HBc positivity. These factors included geographic region and younger age at sexual debut. We also demonstrated that consistent condom use is associated with lower anti-HBc prevalences at the population level.

In a previous study of 1,048 pregnant women between 14 and 19 years in Lima, anti-core prevalence was reported to be 3.5% [21]. Our study, however, reports anti-HBc prevalence among males and females between 18 and 29 years of age from the general population in 28 cities. Similar to other reports [13], [22], anti-HBc prevalence was higher in the jungle than in the highlands and coastal regions. Overall, the reported prevalence of HBV infection is highest in the western Amazon area including Brazil and adjacent regions in Colombia, Peru, and Venezuela [10]. The reason for this finding is not completely clear; however, participants from jungle cities more often reported sexual debut <16 years of age (42.3% for jungle, 19.9% for highlands and 26.2% for coastal cities) and 5 or more lifetime sex partners (25.6% for jungle, 15.8% for highlands and 16.9% for coastal cities).

Lower age at first sexual intercourse was associated with anti-HBc positivity. We found that the odds of anti-HBc positivity increased with younger age at sexual debut, owing to increasing exposure to cumulative risk (10% of protective effect per year of delay of first sexual intercourse according to the multivariable model). Only 4.6% of the participants with anti-HBc positivity also had HBsAg positive results, in accordance with reports of the percentage of HBV infected people having chronic disease [4], [23]. Interestingly, increasing anti-HBc prevalence was found when the analysis was performed according to population at sexual risk, being higher among MSMs than women receiving money or goods for sex or general population. This result is in line with STDs trends in Peru [24], [25].

In the final multivariable model, variables found in previous studies to be associated with HBV infection such as educational level or lifetime number of sex partners were not independently associated with anti-HBc positivity [21], [26], [27]. Among those who reported never having had sexual intercourse, prevalence of anti-HBc was 4.0%, suggesting that sexual activity is not the only mode of hepatitis B transmission in this population. Among those without a sexual history who were anti-HBc positive result, about 75% were between 18 and 21 years indicating early infection, either through vertical or early horizontal transmission.

Male condoms are an efficient and inexpensive way of reducing the risk of acquisition of STIs. As has been demonstrated previously, consistent and correct condom use protects against bacterial STIs [28], including gonorrhea [29], and chlamydial infection [30]. Evidence also confirms that consistent condom use significantly reduces the risk of sexual transmission of HIV [31], herpes simplex virus [32] and human papillomavirus infections [33]. However, data on the effectiveness of condoms in reducing HBV infection have been limited.

In this cross sectional study, we adjusted our crude estimates for variables found to be associated with both condom use and HBV infection: gender, geographic region, and age at sexual debut. In addition, the risk estimate for consistent condom use was significant after adding to the model other potential confounders previously described in the literature such as lifetime number of sex partners and education level [13], [21]. In addition, we were able to estimate the attributable risk percent; but, the OR used for this calculation may be confounded by unmeasured factors, which might affect the estimation of the AR%. Two previous studies have reported that consistent condom use can reduce acquisition of HBV infection among FSW. The first study of acquisition of HBV infection among FSW in India used HBsAg as a serological marker. The intervention group underwent a 6-month program of educational videos, small group discussions, educational materials, received free condoms, and it found incidences of 0.04 compared to 0.12 per person-year of follow-up among intervention and control women, respectively [15]. The second reported that long-term consistent condom use had protective effect for anti-HBc positivity among FSW participating in a control program in Peru [34]. However, two additional studies carried out in other populations found conflicting results. The first, a population-based study in India, used a smaller sample size and found protective effect of condoms only among the female subgroup [35]. The second, from Brazil, found that non-use of condoms was highly frequent (57%) among truck drivers but was not associated with decreased hepatitis B prevalences [36].

Our study has several limitations. First, the questionnaire was designed in the context of a STD intervention and did not include information about non-sexual risk factors for HBV. Although prevalence of intravenous drug use among Peruvians has been considered negligible [37], we could not evaluate other important factors such as tattooing, body piercing, blood transfusions, perinatal transmission, vaccination and household contacts. Second, our survey was limited to a population of 18 to 29 years in urban areas. Information regarding other age groups and rural areas are also essential to understand transmission within the overall population. Third, because condom use was based on self-reports, there is the possibility of incorrect recall or social desirability bias. Our focus on younger adults was in part to reduce recall bias. Fourth, only 40% of the total sample responded questions regarding condom use. We compared demographics and sex-risk variables among those who reported data about condom and those who did not: only distributions of marital status and lifetime number of sex partners were different between both groups (See Supporting Information S4). Thus, inference of results to the general population might not be completely appropriate. Further studies are needed to corroborate our results. Finally, condom use data were based on the participant's last three partners during the last three months and sexual experiences during the past 3 months are not necessarily representative of all sexual experience, raising the possibility of misclassification. However, we believe that any misclassification in the condom use variable would be non-selective with regards to HBV status, and thus would result in attenuation of the true association; as a result, the protective effect of condom could be greater than the association found in this study. The strengths of this study include the random sampling methodologies, the large population-based sample, extensive and explicit data on recent sexual behaviors and sex practices, and the use of computer-assisted self-interview (CASI) techniques to obtain sensitive data.

In conclusion, anti-HBc prevalences are highest in jungle region and among men who have sex with men. There are several risk factors associated with anti-HBc positivity. Consistent condom use was associated with lower prevalences of anti-HBc. Findings from this study emphasize the need for primary prevention programs (vaccination) especially in the jungle population, and imply that condom use promotion could also be a potential strategy to prevent infection with HIV and other sexually transmitted pathogens, but also HBV infection.

## Supporting Information

Supporting Information S1 **Crude results of associations with HBV positivity.** A. Demographic variables associated with HBV positivity. B. Sexual behavior and STI variables associated with HBV positivity. C. Risk factors for HBV infection: bivariate and multivariable model.
(DOC)

Supporting Information S2 **Condom use and HBV infection.** A. Self-reported condom use and HBV infection. B. Association between self-reported condom use and HBV infection by geographic region.
(DOC)

Supporting Information S3 **Association between self-reported condom use and HBV infection by geographic region taking into account sample strata, primary sampling units and population weights.**
(DOC)

Supporting Information S4 **Comparison of variables according to condom use data.** A. Comparison of demographics variables according to condom use data taking into account sample strata, primary sampling units and population weights. B. Comparison of sex risk variables according to condom use data taking into account sample strata, primary sampling units and population weights.
(DOC)
