# Supplementary material for: HBV Infection in Relation to Consistent Condom Use: A Population-Based Study in Peru
Source: PLoS One. 2011 Sep 13;6(9):e24721. doi: 10.1371/journal.pone.0024721 (PMC3172281; doi:10.1371/journal.pone.0024721)
Supplement: Supporting Information S1 — Crude results of associations with HBV positivity. A. Demographic variables associated with HBV positivity. B. Sexual behavior and STI variables associated with HBV positivity. C. Risk factors for HBV infection: bivariate and multivariable model. (DOC) [file pone.0024721.s001.doc]

**SUPPORTING INFORMATION S1**

**A.** Demographic variables associated with HBV positivity

| **Variables** | **Anti-HBc positive/Total** | **p-value** |
| --- | --- | --- |
| ***Gender ^+^*** |  |  |
| Male | 242/3298 (7.34%) | 0.97 |
| Female | 266/3635 (7.32%) |
| ***Geographic region*** |  |  |
| Coastal | 118/3250 (3.63%) | < 0.001 |
| Highlands | 136/2000 (6.80%) |
| Jungle | 258/1750 (14.74%) |
| ***Educational level ^+^*** |  |  |
| ≤11 years | 329/3885 (8.47%) | < 0.001 |
| > 11 years | 161/2894 (5.56%) |
| ***Age at interview ^+^*** |  |  |
| 18 – 21 years | 186/2805 (6.63%) | 0.09 |
| 22 – 25 years | 164/2226 (7.37%) |
| 26 – 29 years | 158/1897 (8.33%) |
| ***Employment ^+^*** |  |  |
| Employed | 187/2402 (7.79%) | 0.26 |
| Unemployed | 318/4515 (7.04%) |
| ***Income ^**, +^*** |  |  |
| No incomes | 251/3479 (7.21%) | 0.87 |
| 1– 500 NS | 185/2538 (7.29%) |
| >500 NS | 62/800 (7.75%) |
| ***Marital status ^+^*** |  |  |
| Never married | 285/4446 (6.41%) | < 0.001 |
| Ever married | 222/2479 (8.96%) |

+Numbers may not add to the total because of missing values.

*Unless stated otherwise: percentages are calculated from row totals

**NS = Nuevos Soles

**B.** Sexual behavior and STI variables associated with HBV positivity ^+, ++^

| **Variables** | **Anti-HBc positive/Total** | **p-value** |
| --- | --- | --- |
| ***Age at sexual debut*** |  |  |
| < 16 years | 171/1589 (10.76%) | < 0.001 |
| 16 – 24 years | 256/4052 (6.32%) |
| 25 – 29 years | 5/83 (6.02%) |
| ***Lifetime number of sex partner*** | |  |
| 0 | 60/980 (6.12%) | 0.003 |
| 1 – 2 | 205/3140 (6.53%) |
| 3 – 4 | 104/1334 (7.80%) |
| 5 or more | 116/1227 (9.45%) |
| ***Last year, new sex partners*** | |  |
| 0 | 227/3345 (6.79%) | 0.19 |
| 1 or more | 85/1066 (7.97%) |
| ***Ever sex with FSW***** | |  |
| No | 134/1834 (7.31%) | 0.82 |
| Yes | 78/1035 (7. 45%) |
| ***Ever sex with MSM***** | |  |
| No | 176/2513 (7.00%) | 0.009 |
| Yes, only insertive sex | 8/73 (10.96%) |
| Yes, only receptive sex | 3/21 (14.29%) |
| Yes, both types of sex | 7/35 (20.00%) |
| ***Received money or other goods for sex (only females)*** | | |
| No | 228/2973 (7.67%) | 0.97 |
| Yes | 4/53 (7.55%) |
| ***Syphilis*** |  |  |
| No | 504/6953 (7.25%) | 0.001 |
| Yes | 8/37 (21.62%) |
| ***HIV infection*** |  |  |
| No | 509/6959 (7.31%) | 0.043 |
| Yes | 3/14 (21.43%) |

+ Numbers may not add to the total because of missing values

++ Analyses included to those with sexual activity (N=5936) except for lifetime number of sex partners

* Unless stated otherwise: percentages are calculated from row totals

** Only male respondents

**C.** Risk factors for HBV infection: bivariate and multivariable model

| **Variables** | **Bivariate model** | **p-value** | **Multivariate model ^+^** | **p-value** |
| --- | --- | --- | --- | --- |
|  | **OR (95%CI)** |  | **OR (95%CI)** |  |
| ***Geographic region*** | | | |  |
| Coastal | 1 (Reference) |  | 1 (Reference) |  |
| Highlands | 1.94 (1.50 – 2.49) | < 0.001 | 2.21 (1.66 – 2.93) | < 0.001 |
| Jungle | 4.59 (3.66 – 5.76) | < 0.001 | 4.30 (3.34 – 5.54) | < 0.001 |
| ***Educational level*** | | | |  |
| ≤11 years | 1 (Reference) |  | 1 (Reference) |  |
| >11 years | 0.64 (0.52 – 0.77) | < 0.001 | 0.62 (0.50 – 0.77) | < 0.001 |
| ***Age at interview **** | | | |  |
| Years (Mean ± SD) | 1.03 (1.01 – 1.06) | 0.01 | 1.05 (1.02 – 1.08) | 0.001 |
| ***Marital status*** | | | |  |
| Never married | 1 (Reference) |  |  |  |
| Ever married | 1.44 (1.20 – 1.72) | < 0.001 |  |  |
| ***Age at sexual debut ^§^*** | | | |  |
| Years (Mean ± SD) | 0.91 (0.88 – 0.94) | < 0.001 | 0.93 (0.90 – 0.97) | < 0.001 |
| ***Lifetime number of sexual partners*** | | | |  |
| 0 | 1 (Reference) |  |  |  |
| 1 – 2 | 1.15 (0.84 – 1.58) | 0.38 |  |  |
| 3 – 4 | 1.13 (0.83 – 1.53) | 0.44 |  |  |
| 5 or more | 1.60 (1.16 – 2.21) | 0.004 |  |  |
| ***Syphilis*** | | | |  |
| No | 1 (Reference) |  |  |  |
| Yes | 3.53 (1.61 – 7.76) | 0.001 |  |  |
| ***HIV infection*** | | | |  |
| No | 1 (Reference) |  |  |  |
| Yes | 3.46 (0.96 – 12.43) | 0.06 |  |  |

+Stepwise logistic regression analysis, adjusted for all demographic and sexual behavioral variables significantly associated with anti-HBc positivity in bivariate analyses (p < 0.10)

***** Per year.

***§*** Per year of delay of first sexual intercourse.
