# Supplementary material for: HBV Infection in Relation to Consistent Condom Use: A Population-Based Study in Peru
Source: PLoS One. 2011 Sep 13;6(9):e24721. doi: 10.1371/journal.pone.0024721 (PMC3172281; doi:10.1371/journal.pone.0024721)
Supplement: Supporting Information S2 — Condom use and HBV infection. A. Self-reported condom use and HBV infection. B. Association between self-reported condom use and HBV infection by geographic region. (DOC) [file pone.0024721.s002.doc]

**SUPPORTING INFORMATION S2**

**A.** Self-reported condom use and HBV infection

| **Variables** | **HBV (-)** | **HBV (+)** | **Model 1*** | **Model 2**** |
| --- | --- | --- | --- | --- |
|  | **n (%)** | **n (%)** | **OR (95%IC)** | **OR (95%IC)** |
| ***Self-reported condom use*** | | |  | |
| Never user | 1059 (91.0) | 105 (9.0) | 1 (Reference) | 1 (Reference) |
| Occasional user | 1248 (92.9) | 95 (7.1) | 0.74 (0.54 – 1.01) | 0.79 (0.58 – 1.09) |
| Consistent user **^§^** | 347 (94.3) | 21 (5.7) | 0.55 (0.33 – 0.93) | 0.66 (0.39 – 1.12) |

§ Consistent condom users were those who reported condom use “always” during last three months with each of their sexual partners

* Adjusted for education level, lifetime number of sexual partners, age at sexual debut, and year of survey.

** Adjusted for geographic region, education level, lifetime number of sexual partners, age at sexual debut, and year of survey.

**B.** Association between self-reported condom use and HBV infection by geographic region

| **Variables** | **Coastal*** | **Highlands*** | **Jungle*** |
| --- | --- | --- | --- |
|  | **OR (95%IC)** | **OR (95%IC)** | **OR (95%IC)** |
| Never user | 1 (Reference) | 1 (Reference) | 1 (Reference) |
| Occasional user | 1.24 (0.64 – 2.43) | 0.77 (0.41 – 1.42) | 0.65 (0.41 – 1.03) |
| Consistent user **^§^** | 0.83 (0.30 – 2.31) | 0.57 (0.20 – 1.57) | 0.68 (0.30 – 1.51) |

* Adjusted for education level, lifetime number of sexual partners, age at sexual debut, and year of survey.
