# Supplementary material for: HBV Infection in Relation to Consistent Condom Use: A Population-Based Study in Peru
Source: PLoS One. 2011 Sep 13;6(9):e24721. doi: 10.1371/journal.pone.0024721 (PMC3172281; doi:10.1371/journal.pone.0024721)
Supplement: Supporting Information S3 — Association between self-reported condom use and HBV infection by geographic region taking into account sample strata, primary sampling units and population weights. (DOC) [file pone.0024721.s003.doc]

**SUPPORTING INFORMATION S3**

**A.** Association between self-reported condom use and HBV infection by geographic region taking into account sample strata, primary sampling units and population weights

| **Self-reported condom use** | **Geographic Region*** | | |
| --- | --- | --- | --- |
|  | **Coast** | **Highlands** | **Jungle** |
|  | **OR (95%IC)** | **OR (95%IC)** | **OR (95%IC)** |
| Never user | 1 (Reference) | 1 (Reference) | 1 (Reference) |
| Occasional user | 0.73 (0.23 – 2.31) | 0.85 (0.36 – 2.04) | 0.65 (0.36 – 1.19) |
| Consistent user | 0.44 (0.13 – 1.58) | 0.32 (0.08 – 1.24) | 0.30 (0.08 – 1.11) |

* Adjusted for gender, education level, lifetime number of sex partners, age at sexual debut, and year of survey.

** Calculations using OR of comparing odds of consistent condom user with odds of never users.
