# Supplementary material for: HBV Infection in Relation to Consistent Condom Use: A Population-Based Study in Peru
Source: PLoS One. 2011 Sep 13;6(9):e24721. doi: 10.1371/journal.pone.0024721 (PMC3172281; doi:10.1371/journal.pone.0024721)
Supplement: Supporting Information S4 — Comparison of variables according to condom use data. A. Comparison of demographics variables according to condom use data taking into account sample strata, primary sampling units and population weights. B. Comparison of sex risk variables according to condom use data taking into account sample strata, primary sampling units and population weights. (DOC) [file pone.0024721.s004.doc]

**SUPPORTING INFORMATION S4**

**A.** Comparison of demographics variables according to condom use data taking into account sample strata, primary sampling units and population weights

| **Variables** | | **With condom data** | **Without condom data** |
| --- | --- | --- | --- |
|  |  | **% (95%CI)** | **% (95%CI)** |
| ***Gender*** | Male | 41.6 (37.4 – 45.9) | 47.3 (43.5 – 51.2) |
|  | Female | 58.3 (54.1 – 62.6) | 52.7 (48.8 – 56.5) |
| ***Geographic region*** | Coastal | 70.6 (68.0 – 73.2) | 69.9 (67.6 – 72.2) |
|  | Highlands | 18.6 (16.7 – 20.4) | 21.1 (19.1 – 23.0) |
|  | Jungle | 10.8 (9.6 – 12.1) | 9.0 (8.0 – 10.0) |
| ***Educational level*** | ≤11 years | 65.0 (61.1 – 68.9) | 63.9 (60.5 – 67.4) |
|  | > 11 years | 35.0 (31.1 – 38.9) | 36.1 (32.6 – 39.5) |
| ***Age at interview*** | 18 – 21 years | 35.5 (31.3 – 39.6) | 35.6 (31.7 – 39.5) |
|  | 22 – 25 years | 35.0 (30.7 – 39.4) | 36.1 (32.3 – 39.8) |
|  | 26 – 29 years | 29.5 (25.0 – 33.9) | 28.4 (24.9 – 31.9) |
| ***Employment*** | Employed | 33.3 (29.3 – 37.3) | 39.1 (35.2 – 43.0) |
|  | Unemployed | 66.7 (62.7 – 70.7) | 60.9 (57.0 – 64.8) |
| ***Income*** | No incomes | 47.7 (42.5 – 52.9) | 46.1 (41.9 – 50.2) |
|  | 1– 500 NS | 39.3 (34.2 – 44.5) | 41.7 (37.6 – 45.9) |
|  | >500 NS | 13.0 (10.2 – 15.8) | 12.2 (9.9 – 14.5) |
| ***Marital status*** | Never married | 49.7 (45.3 – 54.0) | 61.8 (58.1 – 65.4) |
|  | Ever married | 50.3 (46.0 – 54.7) | 38.2 (34.6 – 41.9) |

**B.** Comparison of sex risk variables according to condom use data taking into account sample strata, primary sampling units and population weights

| **Variables** | | **With condom data** | **Without condom data** |
| --- | --- | --- | --- |
|  |  | **% (95%CI)** | **% (95%CI)** |
| ***Age at sexual debut*** | < 16 years | 24.4 (20.3 – 28.4) | 28.5 (25.0 – 32.0) |
|  | 16 – 24 years | 74.7 (70.6 – 78.8) | 70.1 (66.6 – 73.6) |
|  | 25 – 29 years | 0.9 (0.5 – 1.4) | 1.4 (0.7 – 2.1) |
| ***Lifetime number of sex partners*** | 1 – 2 | 61.5 (57.2 – 65.8) | 50.2 (46.1 – 54.3) |
|  | 3 – 4 | 19.3 (16.0 – 22.6) | 27.7 (24.2 – 31.2) |
|  | 5 or more | 19.2 (15.5 – 22.8) | 22.1 (18.9 – 25.3) |
| ***Last year, new sex partners*** | 0 | 77.7 (73.9 – 81.6) | 74.7 (70.6 – 78.8) |
|  | 1 or more | 22.3 (18.4 – 26.1) | 25.3 (21.2 – 29.4) |
| ***Ever sex with FSW*** | No | 69.5 (64.0 – 74.9) | 63.4 (57.8 – 69.0) |
|  | Yes | 30.5 (25.1 – 36.0) | 36.6 (31.0 – 42.2) |
| ***Ever sex with MSM*** | No | 95.2 (93.6 – 97.7) | 95.2 (92.8 – 97.7) |
|  | Yes, only insertive | 3.1 (1.2 – 5.1) | 2.1 (1.0 – 3.2) |
|  | Yes, only receptive | 0.6 (0.2 – 1.1) | 0.4 (0.0 – 9.5) |
|  | Yes, both types | 0.6 (0.2 – 1.0) | 2.2 (0.1 – 4.4) |
| ***Received money or other goods for sex*** | No | 97.2 (95.8 – 98.5) | 96.2 (94.6 – 97.9) |
|  | Yes | 2.8 (1.5 – 4.2) | 3.8 (2.1 – 5.4) |
| ***Syphilis*** | No | 99.9 (99.8 – 100.0) | 99.1 (98.2 – 100.0) |
|  | Yes | 0.1 (0.0 – 0.2) | 0.9 (0.0 – 1.8) |
| ***HIV infection*** | No | 99.8 (99.6 – 100.0) | 99.8 (99.5 – 100.0) |
|  | Yes | 0.2 (0.0 – 0.4) | 0.2 (0.0 – 0.5) |
